# The HRASLS (PLA/AT) subfamily of enzymes

**DOI:** 10.1186/s12929-015-0210-7

**Published:** 2015-10-26

**Authors:** Emily B. Mardian, Ryan M. Bradley, Robin E. Duncan

**Affiliations:** Department of Kinesiology, University of Waterloo, BMH 2415, Waterloo, ON N2L 3G1 Canada; Department of Kinesiology, University of Waterloo, BMH 1110, Waterloo, ON N2L 3G1 Canada

**Keywords:** H-RAS-like suppressor enzymes, Acyltransferase, Phospholipase A_1/2_, Class II tumour suppressor, N-acylphosphatidylethanolamine (NAPE), Pla2g16, TIG3, RARRES3, RIG1, iNAT

## Abstract

The H-RAS-like suppressor (HRASLS) subfamily consists of five enzymes (1–5) in humans and three (1, 3, and 5) in mice and rats that share sequence homology with lecithin:retinol acyltransferase (LRAT). All HRASLS family members possess in vitro phospholipid metabolizing abilities including phospholipase A_1/2_ (PLA_1/2_) activities and O-acyltransferase activities for the remodeling of glycerophospholipid acyl chains, as well as N-acyltransferase activities for the production of N-acylphosphatidylethanolamines. The in vivo biological activities of the HRASLS enzymes have not yet been fully investigated. Research to date indicates involvement of this subfamily in a wide array of biological processes and, as a consequence, these five enzymes have undergone extensive rediscovery and renaming within different fields of research. This review briefly describes the discovery of each of the HRASLS enzymes and their role in cancer, and discusses the biochemical function of each enzyme, as well as the biological role, if known. Gaps in current understanding are highlighted and suggestions for future research directions are discussed.

## Introduction

**Fig. 1:**
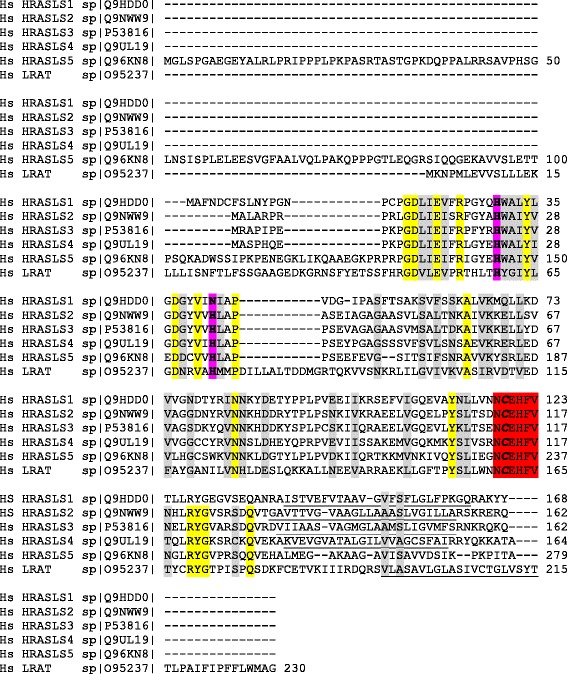
Sequence alignment of human HRASLS enzymes. Sequence alignment was performed using ClustalW2 [51]. The C-terminal transmembrane-spanning hydrophobic region is underlined. The NCEHFV motif is highlighted in red with the active site cysteine bolded and italicized. The remaining two active sites in the catalytic triad are also bolded, and are highlighted in purple. Conserved residues are highlighted in yellow, and homologous residues are highlighted in grey

The H-RAS-like suppressor (HRASLS) enzymes consist of five members (1–5) in humans and three (1, 3, and 5) in mice and rats [1, 2]. Also known as lecithin:retinol acyltransferase (LRAT)-like proteins due to their sequence homology to LRAT (Fig. 1) [3], the HRASLS enzymes comprise a vertebrate subfamily of the papain-like or NlpC/P60 thiol proteases that includes LRAT [4]. Enzymes in this superfamily have diverse functions, forming a conserved group of homologous proteins found broadly in the bacterial superkingdom, in DNA and positive strand RNA viruses, in archaea, and in eukaryotes [4].

**Fig. 2:**
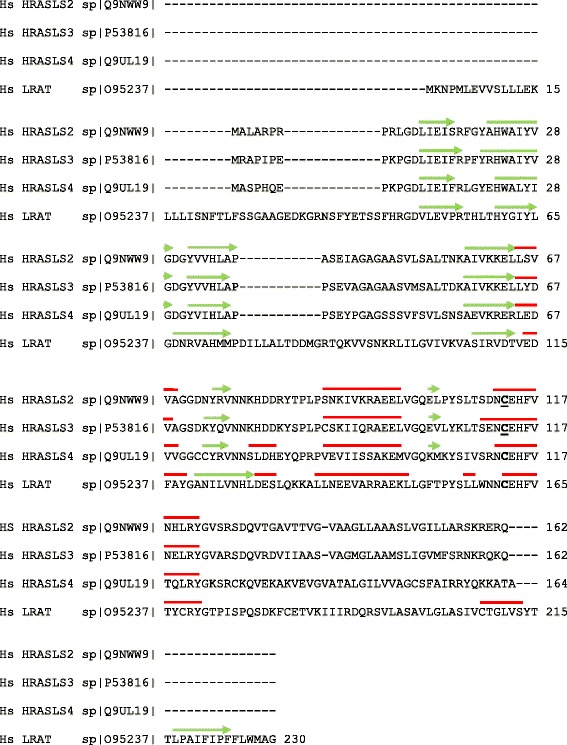
Structural alignment of human HRASLS enzymes. Sequence alignment was performed using ClustalW2 [51] and structural alignment was performed for HRASLS2, HRASLS3, and HRASLS4 as described by Golczak *et al.* in 2012 [3] and Wei *et al*. in 2015 [6]. The β-sheets are indicated by green arrows and α-helices are indicated by red lines

**Fig 3:**
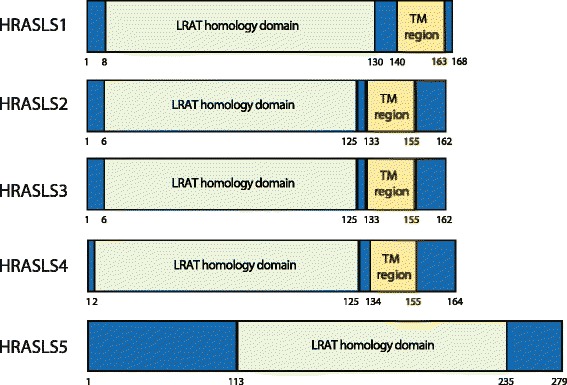
Homology domains within the five human HRASLS enzymes. LRAT (lecithin:retinol acyltransferase) homology domain and the predicted transmembrane region (TM region) are shown

HRASLS family members share several common elements (Figs. 1, 2 and 3), including a highly conserved NCEHFV sequence in the C-terminal region and similar basic structural motifs [2, 3]. Several residues in the NCEHFV sequence are critical for acylation and deacylation reactions, including the cysteine that acts as the active site nucleophile [2]. This residue is paired in HRASLS 2–5 with two N-terminal region histidines, and in HRASLS1 with N-terminal region histidine and asparagine residues, forming an unconventional catalytic triad that is characteristic of NlpC/P60 family members [4]. In this arrangement, the first histidine acts as a base to deprotonate the sulfhydryl group of the cysteine side chain, while the second polar residue acts to stabilize the correct orientation of the imidazole ring of the first histidine [2]. With the exception of HRASLS5, these proteins also contain a C-terminal transmembrane-spanning hydrophobic domain for endomembrane localization (Fig. 1) [5]. Currently, only the structures of HRASLS2, HRASLS3, and HRASLS4 have been reported. The crystal structures of HRASLS2 and HRASLS3 were solved by Golczak *et al*. in 2012. They were both shown to contain three α-helices well separated from a four-stranded antiparallel β-sheet, which resembles the basic structure of the NlpC/P60 thiol proteases that contain an α + β fold with segregated αβ segments (Fig. 2) [3, 4]. The NMR structure of HRASLS4 was solved by Wei *et al*. in 2015 and showed similar folding to that of HRASLS3, although the basic structure contained four α-helices and a six-stranded antiparallel β-sheet (Fig. 2) [6].

**Fig 4:**
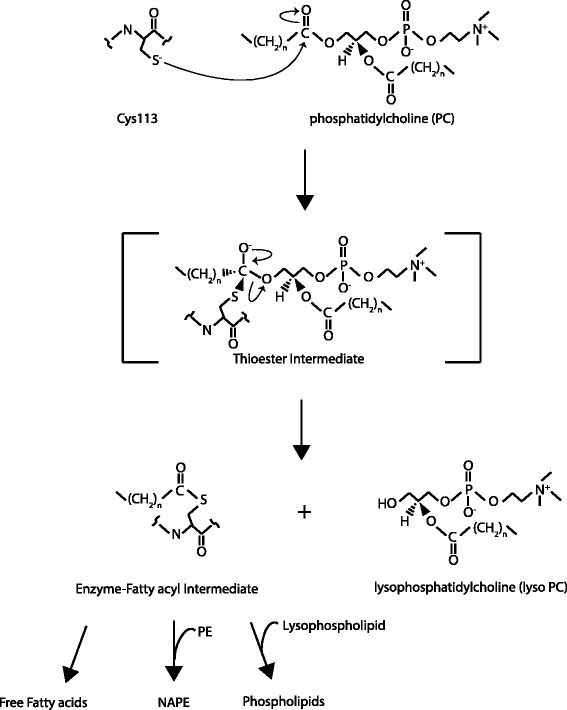
Illustration of a portion of the catalytic mechanism of HRASLS enzymes including depiction of the thioester intermediate formed during phospholipid hydrolysis. Reaction demonstrating the formation of a thioester intermediate following nucleophilic attack by the anionic sulphur of the deprotonated active site cysteine side chain in HRASLS proteins on the carbonyl group of the *sn-1* fatty acyl chain of phosphatidylcholine. Hydrolysis results in the generation of a free lysophospholipid and an enzyme-fatty acyl intermediate that releases the enzyme and a free fatty acid following addition of a hydroxyl group to the carbonyl of the fatty acyl chain, forming a carboxylic acid. All HRASLS enzymes can catalyze complete phospholipase reactions in vitro, resulting in liberation of a free fatty acid as well as a lysophospholipid. All HRASLS enyzmes also show in vitro N- and O-acyltransferase activities, where the enzyme-fatty acyl intermediate binds a glycerolphospholipid leading to production of N-acylphosphatidylethanolamines (NAPEs) or phospholipids, respectively

Recent evidence has indicated a biochemical role for all HRASLS homologues in acyl chain remodeling of glycerophospholipids [3]. Using mass spectrometry analysis, a thioester intermediate has been detected to form between cysteine 161 of purified LRAT and the acyl side-chain donated by phosphatidylcholine (PC) [7]. This suggests that the transient acylation of a reactive cysteine residue also occurs in the LRAT-like HRASLS enzymes to mediate reactions involving glycerophospholipids (Fig. 4) [3]. HRASLS1-5 all possess in vitro Ca^2+^ independent phospholipaseA_1/2_ (PLA_1/2_) activities, which requires a pH of 8–9, dithiothreitol (DTT), and Nonidet P-40 for maximum function, and which are inhibited by iodoacetate [8–10]. The importance of DTT in particular suggests that preventing oxidation of sulfhydryl groups is critical for maintaining enzyme stability, which agrees with a predicted role for an active site cysteine in HRASLS enzymes [8]. Both PC and phosphatidylethanolamine (PE) act as substrates with PLA_1_ activity showing dominance over PLA_2_ activity for HRASLS1, 2, 4, and 5 [2, 8–11]. In regards to HRASLS3, there has been contradictory evidence of substrate specificity and positional preference for acyl hydrolysis, as well as other enzymatic characteristics [12–15]. Uyama *et al*. reported both PLA_1/2_ activities with PC and PE as substrates, but a preference for hydrolysis at the *sn*-1 position [15]. Duncan *et al*. also found that this enzyme shows both PLA_1/2_ activities with maximal phospholipase activity at pH 8, and they further identified a broad range of possible substrates, showing evidence of acyl hydrolase activity by HRASLS3/AdPLA against not only PC and PE, but also phosphatidylserine, and phosphatidylinositol, but not phosphatidic acid [14]. However, in contrast to the findings of Uyama *et al.*, Duncan *et al.* reported a preference for hydrolysis by this enzyme at the *sn*-2 position that is moderately stimulated by Ca^2+^ [14]. The inconsistencies within the literature have been proposed to be due to differences in the assay systems used [15]. Based on the evidence to date, it seems that this enzyme should best be considered a PLA_1/2_ [14–16].

**Fig 5:**
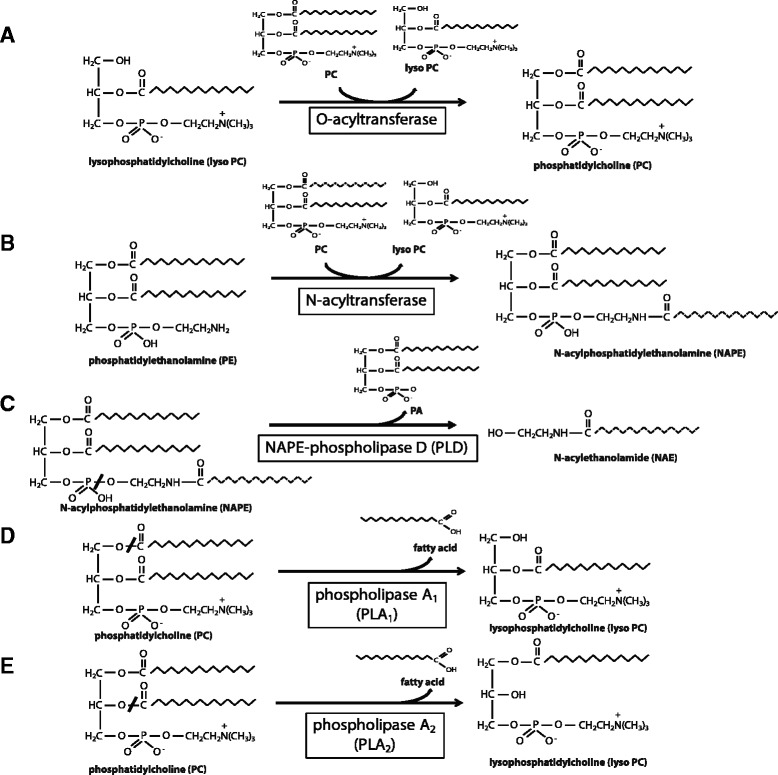
Examples of phospholipid metabolism reactions involving HRASLS enzymes. *A*, O-acyltransferase (acyl-CoA independent transacylase) reaction demonstrating the conversion of lyso PC to PC, using the *sn-1* acyl chain of PC as the acyl donor. *B,* N-acyltransferase reaction demonstrating the acylation of the amino group of phosphatidylethanolamine using the *sn-1* fatty acyl chain donated by PC, resulting in the generation of an N-acylphosphatidylethanolamine (NAPE) and a lyso PC. *C,* Conversion of NAPE to N-acylethanolamine (NAE) by NAPE-phospholipase D-mediated hydrolysis. *D,* Phospholipase A_1_ (PLA_1_) reaction demonstrating the cleavage of a fatty acyl chain from the *sn-1* position of PC, resulting in the liberation of a free fatty acid and a 1-hydroxy-2-acyl-lyso PC. *E,* Phospholipase A_2_ (PLA_2_)-mediated conversion of PC to lyso PC by hydrolysis of a fatty acyl chain at the *sn-2* position

The HRASLS family of proteins also possess O-acyltransferase activity, which describes the acyl-CoA independent transacylation of a lysophospholipid at either the *sn-1* or *sn-2* position using a fatty acyl chain donated by a phospholipid (Fig. 5) [1–3]. Each member of the HRASLS family has shown some preference for O-acylation of lysophosphatidylcholine (lyso PC) at the *sn*-1 position [1, 8, 10]. It has therefore been proposed that the HRASLS family be reclassified as a novel group of phospholipid metabolizing enzymes, respectively known as phospholipases A/acyltransferases (PLA/AT) 1–5 [1].

The HRASLS family of proteins also function in vitro to differing extents as N-acyltransferases for the production of N-acylphosphatidylethanolamines (NAPEs). HRASLS1-5 have been shown to catalyze the transfer of an acyl chain from the *sn*-1 position of a glycerophospholipid to the amino group of PE producing NAPEs, which serve as precursors for N-acylethanolamines (NAEs) (Fig. 5) [1]. NAEs are a class of bioactive signalling molecules produced when a NAPE molecule is cleaved through hydrolysis, as catalyzed by NAPE-hydrolyzing phospholipase D (NAPE-PLD) (Fig. 5). NAEs have myriad biological roles, including modulation of inflammation, energy regulation, and nociception [1, 17]. Although little is yet known regarding the physiological significance of NAE regulation by HRASLS subfamily members, these enzymes are already known to play diverse biological roles in vivo. In particular, a function has been investigated for several members as class II tumour suppressor genes. Class II tumour suppressors are involved in the negative regulation of cell growth, and are often down-regulated in cancer cells secondary to other genetic or metabolic changes, but are not themselves mutated or deleted. As a result, they make attractive therapeutic targets, since they should be inducible if appropriate conditions or agents are identified [18–20]. Due to the study of HRASLS subfamily members in wide ranging fields, rampant renaming has occurred, with the result that there are now 27 different designations for these five enzymes (Table 1). Here, we identify and review the biochemical and biological role of these enzymes, and seek to disambiguate the current state of knowledge on this enzyme subfamily.

**Table 1:** Aliases for the HRASLS family of enzymes

	HRASLS-1	HRASLS-2	HRASLS-3	HRASLS-4	HRASLS-5
Aliases	A-C1	PLA_1/2_-2	HRSL3	HRSL4	HRSL5
	HRASLS	PLA/AT-2	HREV107	TIG3	RLP-1
	HRSL1		HREV107-1	RARRES3	HRLP5
	PLA/AT-1		H-REV107-1	RIG1	iNAT
			HREV107-3	PLA_1/2_-3	PLA/AT-5
			PLA_2_G16	PLA/AT-4	
			MCG118754		
			RLP-3		
			adPLA		
			PLA/AT-3		
Chromosomal location	Human 3q29	Human 11q12.3	Human 11q12.3	Human 11q23	Human 11q13.2
	Mouse 16 B2		Mouse 19 A		Mouse 19 A
NM accession No. (GenBank)	Human NM_020386.4	Human NM_017878.1	Human NM_001128203.1	Human NM_004585.4	Human NM_001146728.1
	Mouse NM_013751.5		Mouse NM_139269.2		Mouse NM_025731.2
NP accession No. (GenBank)	Human NP_065119.2	Human NP_060348.1	Human NP_001121675.1	Human NP_004576.2	Human NP_001140200.1
	Mouse NP_038779.2		Mouse NP_644675.2		Mouse NP_080007.1

## Review

### HRASLS1 (PLA/AT1)

HRASLS1 was originally discovered by Akiyama *et al.* in 1999 through differential display between two mouse cell lines [21]. The 167-amino acid long protein was named A-C1, and was found to be 46 % homologous with a rat tumour suppressor protein called rat H-rev107 (now also called HRASLS3, among other names (see Table 1)) [11, 21]. The 168 aa-long human *HRASLS1/A-C1* gene was also cloned, and was found to share 83 % identity with the mouse sequence [8, 22]. A tumour suppressor role for HRASLS1/A-C1 was tested directly and it was found that, indeed, overexpression of HRASLS1/A-C1 inhibited the growth of *H-Ras*-transformed NIH3T3 fibroblasts [21]. Additionally, expression of the human homolog was found to be reduced in gastric cancers from 41 different individuals due to hyper-methylation of CpG islands in the 5’ region of the *HRASLS1* gene, suggesting that it also plays a role as a tumour suppressor in human disease [23].

The enzymological role was first assessed over a decade after HRASLS1 was cloned [8]. Reports demonstrate a ratio of 1.6 [1] or higher [24] for human, mouse and rat HRASLS1/AC-1 PE N-acyltransferase activity to PLA_1/2_ activity, indicating greater specific N-acyltransferase activity in vitro [1, 24]. This is supported by metabolic labelling experiments in cells [1, 2, 8, 24].

The tissue distribution of HRASLS1 is distinguishable from other members of the HRASLS family of enzymes. In humans, mice, and rats, HRASLS1/A-C1/PLA/AT1 is predominately expressed in testes, skeletal muscle, and heart [8]. Humans show relatively low expression of HRASLS1 in most other tissues [8], while mice and rats also show abundant expression in brain, with some evidence of expression in thymus, lung, stomach, kidney, and bone marrow cells [8, 21]. N-acylethanolamines (NAEs) are known to accumulate in response to acute injury in tissues, including the heart, brain, and testicles, suggesting a possible role for HRASLS-1 in mediating pathological events in these tissues [17]. Thus far, however, no studies have examined the physiological role of HRASLS1.

### HRASLS2 (PLA/AT2)

HRASLS2 was first cloned from SW480 colon cancer cells [5]. This member of the HRASLS family is located on chromosome 11 in humans, but is lacking from the rodent genome [5, 10]. The HRASLS2 gene transcript is detectable primarily in gastrointestinal tissues, including the colon, small intestine, and stomach, and is also expressed in the trachea and kidney [5]. Subcellular localization of HRASLS2 is primarily within the perinuclear region, where it shows a granular pattern [5], similar to that visualized for HRASLS1 [21].

A role for HRASLS2 in cancer and, specifically, in RAS-mediated cellular transformation, has been investigated. Transfection of HRASLS2 into HCT166 colon cancer cells, HeLA cervical cancer cells, and MCF-7 breast cancer cells was shown to suppress colony formation [5]. As well, HRASLS2 over-expression suppressed the activation of wildtype and mutant RAS in HtTA cervical cancer cells, resulting in increased cell death [5]. These effects were dependent on the C-terminal hydrophobic domain. Truncation of the last 26 amino acids containing this region eliminated the pro-apoptotic, anti-RAS, and growth suppressive activities of HRASLS2 [5]. In addition, loss of the putative C-terminal transmembrane domain also caused HRASLS2 to translocate, demonstrating increased intensity in the nucleus, which highlights the importance of this region in endomembrane localization [5].

The phospholipid metabolizing activities of HRASLS2 have been assessed using both cell homogenates and purified enzyme. HRASLS2 overexpression in COS-7 cells was found to increase in vitro PLA_1/2_ activity, with similar increases evident in assays conducted using either post-microsomal supernatants or microsomal pellets [10]. HRASLS2 was also shown to prefer to use fatty acids from the *sn*-1 position of PC as acyl donors for its N-acyltransferase activities [1, 10]. In vitro, N-acyltransferase activity was found to be approximately 4-fold greater than PLA_1/2_ activity [1, 10]. Because of the preference for activity at the *sn-1* position, the major N-acyl species of NAPE produced by the overexpression of HRASLS2 in COS-7 cells tended to contain mostly saturated and monounsaturated acyl chains, with N-palmitoylethanolamide, N-stearoylethanolamide, and N-oleoylethanolamide forming the predominant NAEs generated, while N-arachidonoylethanolamide (i.e. anandamide), and its precursor N-arachidonoyl PE, showed only minimal elevation [1].

The physiological function of HRASLS2 is as-of-yet uncharacterized. Overexpression of HRASLS2 in clonal cell lines has been shown to decrease endogenous plasmenylethanolamine levels, which causes abnormal localization of peroxisomal proteins [1]. A similar result has also been observed for HRASLS3 [12]. Since the predominant activity of that enzyme is PLA_1/2_ rather than N-acyltransferase activity, it is unlikely that changes in NAPE/NAE levels or synthesis play a role in mediating peroxisomal events by HRASLS subfamily members. Further studies with *Hrasls2* gene ablation will be important to elucidate the biological role of this enzyme.

#### HRASLS3 (PLA/AT3)

Diverse studies on both the biological role and enzymatic function of HRASLS3 have led to the generation of a particularly large number of aliases, with at least ten different names recorded in the literature (Table 1) [9, 13, 25]. HRASLS3 was the first of the HRASLS subfamily of proteins to be discovered [11]. It was identified by subtractive hybridization performed between *H-ras* transformed rat fibroblasts and phenotypic revertants that had regained density-dependent growth inhibition, and thus was initially given the name *H-ras* revertant #107 (H-rev 107) to denote this [11]. In additional studies, HRASLS3/H-rev107 was categorized as a class II tumour suppressor [11, 26]. It has been suggested that HRASLS3 regulates oncogenic *H-ras* through its ability to decrease cellular levels of ether-type lipids, such as plasmalogens and monoalkyldiacyglycerols, by decreasing cellular levels of peroxisomes that are major sites for the synthesis of these lipids [12, 27]. Uyama *et al.* found that HRASLS3 binds to the peroxisomal chaperone protein Pex19p through its C-terminal and N-terminal hydrophobic domains [27]. Subsequently, Pex19p is inhibited from binding to peroxisomal membrane proteins such as Pex3p and Pex11βp, which results in decreased peroxisomal biogenesis [27]. Additional studies, however, are still required to directly test the relative importance of peroxisomal biogenesis regulation in mediating effects of HRASLS3 on the *H-ras*-dependent growth of cancer cells.

HRASLS3 is downregulated in many cancers by methylation of the CpG-rich region in the promoter, which results in gene silencing [18, 28, 29]. In OVCAR-3 ovarian cancer cells, HRASLS3 has been show to interact through its N-terminal proline-rich domain to ablate the catalytic activity of protein phosphatase 2A [25], causing caspase-9-dependent cell death. As well, HRASLS3 was found to inhibit cell migration and invasion when expressed in human NT2/D1 testicular cancer cells, and this function was suggested to relate to its ability to enhance the activity of prostaglandin D2 synthase [30]. At least one study has demonstrated, using chemical inhibitors, that the phospholipase activity of HRASLS3/H-rev107 is required for its anti-RAS effect [31]. This is also in agreement with an earlier finding that the growth-inhibitory and tumor-suppressing activity of HRASLS3/H-rev107 requires the C-terminal region [26] that is required for catalysis [16].

Not all studies, however, have found an inhibitory role for HRASLS3 in cancer. HRASLS3 does not inhibit, but instead stimulates, the proliferation of non-small cell lung carcinomas, contributing to tumour progression [29]. *HRASLS3* has also been found to be a downstream target of mutant p53, with increased *HRASLS3* levels reported in p53 mutant osteosarcomas [32]. As well, increased proliferation, migration, and invasion of osteosarcoma cells was reported following overexpression of *HRASLS3* [32]*.* Additional studies on HRASLS3 active site mutants in transformed cell lines will help to isolate its lipid-enzymatic function from its function in tumour growth modulation.

The enzymatic activity of HRASLS3/RLP-3 was first investigated in 2007 by Jin *et al.*, who demonstrated N-acyltransferase activity for this enzyme in vitro [9]. Subsequent work indicated the predominance of PLA over N- or O-acyltransferase activities for HRASLS3 in vitro [1, 8], and a detailed characterization of this enzyme as an adipose-specific phospholipase A_2_ (AdPLA) has been performed [14]. On the basis of studies performed using general phospholipase chemical inhibitors, it was suggested that this enzyme constituted the first member of an entirely new group of phospholipases A_2_, group XVI. As a result, the gene was subsequently renamed PLA2G16 [14].

Highly detailed analyses of human HRASLS3 have been performed in a series of elegant papers, including X-ray crystallographic analysis of its structure [3, 16, 33]. In the first structure analysis of the HRASLS family of proteins, Ren *et al.* demonstrated in 2010 that the phospholipase active site of HRASLS3 contains a Cys^113^-His^23^-His^35^ catalytic triad [33]. This finding was supported by Pang *et al.* in 2012 who showed that the phospholipase activity of HRASLS3 was mediated by this catalytic triad [16]. As is also seen in LRAT-NlpC/P60 family proteins, the active-site cysteine performs a nucleophilic attack on the *sn-*1 and *sn-*2 acyl groups of phospholipids. In this reaction, the pK_a_ of Cys^113^ is too high to allow it to act as a nucleophile alone [16]. Thus, His^23^ assists in deprotonating the sulfhydryl side chain of Cys^113^, which effectively lowers the pK_a_ from 8.3-8.8 to 7.0, allowing it to function in the deacylation of phospholipids. This study also provided mechanistic insight into the higher phospholipase activity relative to acyltransferase activity of HRASLS3 in particular. Evaluation of hydrogen/deuterium exchange at the active site of HRASLS3 suggested that during catalysis, water could readily access reactants, allowing the reactive intermediate to decompose to a free fatty acid and a lysophospholipid [16]. It remains to be determined whether varying degrees of hydrophobicity at the active site are related to the acyltransferase/phospholipase activity ratio of other HRASLS subfamily members. The C-terminal hydrophobic domain is critical for HRASLS3 activity. Truncation of the C-terminal hydrophobic domain results in loss of HRASLS3 from membranes, and a loss of HRASLS3-mediated phospholipase activity in vitro, indicating the critical nature of this hydrophobic span for interfacial catalysis [27].

The in vivo role of HRASLS3 has been studied in some depth [13]. HRASLS3/AdPLA is expressed predominantly in white adipose tissue (WAT), and also to a lesser extent in brown adipose tissue [13, 14], where it is a major regulator of lipolysis and is crucial for the development of obesity, as seen in an *Hrasls3*^*−/−*^*/AdPLA*^*−/−*^ mouse model [13]. The ablation of *Hrasls3/AdPLA* causes a reduction in adipose tissue mass and triacylglycerol content, despite normal adipogenesis, as a result of constitutively elevated rates of lipolysis. The underlying molecular mechanism involves the regulation of lipolysis through the modulation of prostaglandin E_2_ (PGE_2_) levels and signaling. HRASLS3/AdPLA is responsible for 80 % of adipocyte phospholipase activity, which is a major source of arachidonic acid that is used in the synthesis of eicosanoids [13]. As a result, loss of HRASLS3/AdPLA results in a dramatic fall in levels of PGE_2_ [13]. The primary receptor for PGE_2_ in adipocytes is prostaglandin E receptor 3 (EP3), which is unique among EPs in that it is Gα_i_–coupled, and therefore exerts a repressive action on adenylyl cyclase in adipocytes when activated [34]. Thus, loss of HRASLS3/AdPLA in mice causes a decrease in PGE_2_ levels and concomitant reduced activation of inhibitory EP3, with the result that generation of cAMP, activation of the hormone-sensitive lipase, and stimulation of lipolysis proceeds unchecked, preventing the accumulation of triacylglycerol and the development of genetic or diet-induced obesity [13]. Since HRASLS3/AdPLA is normally stimulated by insulin, increased activity by this enzyme may help to modulate the antilipolytic effects of insulin in adipocytes in the fed state [13]. The role of HRASLS3/AdPLA in adipocyte triglyceride metabolism suggests that inhibition may be a useful strategy to prevent and treat obesity [13, 16], and future studies will therefore likely focus on the design of inhibitors [16] and on strategies to regulate this enzyme.

### HRASLS4 (PLA/AT4)

HRALS4 was identified by three separate groups in 1998 [35], 2000 [36], and 2001 [37] as a retinoid-inducible anti-proliferative/class II tumour suppressor gene that goes by the alternate names Tazarotene-induced protein 3 (TIG3), Retinoic acid receptor responder protein 3 (RARRES3), and Retinoid-inducible gene 1 (RIG1) (see Table 1 for all aliases). HRASLS4 is homologous with HRASLS3/H-rev107 [35, 36] and, like that enzyme, has reduced expression in a wide variety of primary human tumours, including lymphoma, ureter, kidney, rectal, and uterine, and cancer cell lines, including HL-60 promyelocytic leukemia cells, HeLa cells, K-562 chronic myelogenous leukemia cells, SW480 colon carcinoma, A549 colon carcinoma, and G361 melanoma cells [35]. HRASLS4 also shows growth inhibitory effects when overexpressed in T47D Chinese hamster ovary cells [35]. A role for HRASLS4 in malignant disease continues to be studied extensively, and a comprehensive overview of this area is beyond the scope of the current review. However, it is of interest to mention some of the more recent findings. The HRASLS4 promoter contains a response element for the p53 tumour suppressor that is activated by wildtype but not mutant p53 [38]. Whether this feature exists for all HRASLS subfamily members has not yet been studied, but it would help to explain the almost universal down-regulation of these proteins in transformed cells.

Like other HRASLS subfamily members, HRASLS4 also inhibits H-RAS-mediated signalling [5, 39, 40]. The anti-cancer activity of HRASLS4 has been localized specifically to its action within the Golgi. While the C-terminal hydrophobic domain of HRASLS4 anchors this enzyme within both the endoplasmic reticulum and the Golgi apparatus, only Golgi-targeted HRASLS4 induces apoptosis in cancer cells [39]. The phospholipid metabolizing activity of HRASLS4 appears to be important for its anti-cancer effects, particularly with regards to metastasis and invasion [41]. Similar to HRASLS3, HRASLS4 functionally interacts with prostaglandin D_2_ synthase to augment the production of prostaglandin D_2_ [42]. This function was found to be dependent on an intact C-terminal hydrophobic domain, and although not tested directly, it seems highly likely to be related to the phospholipase activity of this enzyme [42].

A role for HRASLS4 in phospholipid metabolism has been investigated [1, 10]. HRASLS4 functions in vitro as a Ca^2+^ independent PLA_1/2_ [1, 8, 10]. Although N-acyltransferase activity in vitro has been shown to be minor or absent for this enzyme [10], HRASLS4 does significantly increase the cellular content of both NAPE and NAE in metabolic labelling experiments [1]. The functional significance of this activity with regards to the anti-cancer activity of this enzyme remains to be determined.

A physiological role for *Hrasls4* has been investigated [43]. HRASLS4 is found in the suprabasal epidermis of skin, where it interacts with and activates another enzyme, transglutaminase I (TG1), which functions during terminal differentiation to form covalent bonds between proteins at the inner surface of the plasma membrane [44]. The action of TG1 is critical for production of the cornified envelope that maintains the epidermal barrier [44], and adenoviral delivery of HRASLS4 to cultured keratinocytes results in the activation of TG1, resulting in differentiation-like cell death, as well as the generation of cornified envelope-like structures [45, 46]. Reduced expression of HRASLS4 is evident in hyperproliferative conditions such as psoriasis [47]. An additional role for HRASLS4 in keratinocyte differentiation is suggested by findings that this protein also localizes to the centrosome, where it affects microtubule kinetics and cell division [43, 48]. Interaction between the centromere and HRASLS4 has been found to involve a central region of the enzyme that contains the NCEHFV sequence [43]. However, whether the phospholipid metabolizing abilities of this enzyme, *per se*, are critical for its various physiological roles in skin cells, remains to be clearly characterized.

### HRASLS5 (PLA/AT5)

HRASLS5 was first investigated as part of a search for new Ca^2+^-independent enzymes active in the N-acylation of PE [9] (also called RLP-1 and iNAT; see Table 1 for all aliases). HRASLS5 displays dominate N-acyltransferase activity over both PLA_1/2_ and O-acylation activities in vitro, and overexpression of HRASLS5 enhances formation of NAPE and NAE in cultured cells [1, 2, 8, 9]. However, HRASLS5 differs from other HRASLS family members in that it is predominantly present in the cytosol, likely due to the absence of the C-terminal hydrophobic span that is characteristic of HRASLS enzymes 1–4 (Figs. 1, 2, 3 and 4) [1, 9]. HRASLS5 also does not show a clear preference for abstraction of acyl groups from either the *sn*-1 or *sn*-2 positions of PC during N-acylation reactions [9]. It has been suggested that HRASLS5 may be involved in the production of anandamide (arachidonoyl ethanolamide) because of its capacity to utilize fatty acyl chains from the *sn-1* or *sn-2* position of phospholipids [9]. However, studies have yet to characterize the nature of endogenous NAPE and NAE species produced by HRASLS5. Likewise, little is known regarding the physiological function of this enzyme. It has been identified in spermatocytes of developing rat testes, but its function in that tissue is currently unknown [49]. Similarly, studies have yet to investigate whether it has a role in cancer like the other HRASLS family members.

## Conclusions

The five members of the HRASLS family of enzymes have been shown to have phospholipase A_1/2_ activity, and O- and N-acyltransferase activity. In addition, studies generally report reduced expression of these enzymes in cancer cells, and demonstrate a direct anti-cancer role, although not all studies agree. Evidence from studies using mutated or truncated forms of HRASLS subfamily members suggests that the phospholipid metabolizing functions are required for some, but likely not all, of the effects observed in tumour cells. Further work should integrate recent advances in understanding the biochemical function of these enzymes to better understand mechanisms related to aberrant cell growth in neoplasia. Further studies should also focus on understanding the major physiological function of each homologue in cells and tissues, using recent advances in gene editing techniques as well as the generation of gene knockout mice. Human monozygotic twins with Poland Syndrome have been found to be heterozygous for a gene deletion event on chromosome 11 that removes *HRASLS2-5,* strongly suggesting a role for one or more of these genes in causation of this disorder [50]. Poland Syndrome is characterized by hypoplasia/aplasia of the pectoralis major muscle and other variable anomalies including hypoplasia/aplasia of mammary tissue and ribs, limited subcutaneous fat, and sternal anomalies [50]. Generation of gene knockout mice will be required to understand any putative role for this enzyme subfamily in normal physiology and in human disease.

## Abbreviations

AdPLA: adipose-specific phospholipase A_2_
DTT: dithiothreitol
HRASLS: H-RAS-like suppressor
LRAT: lecithin:retinol acyltransferase
lyso-PC: lysophosphatidylcholine
NAPE: N-acylphosphatidylethanolamine
NAPE-PLD: N-acylphosphatidylethanolamine-hydrolyzing phospholipase D
NAE: N-acylethanolamines
PLA/AT: phospholipases A/acyltransferases
PC: phosphatidylcholine
PE: phosphatidylethanolamine
PLA: phospholipase A

## References

[1] Uyama T, Ikematsu N, Inoue M, Shinohara N, Jin XH, Tsuboi K (2012). Generation of N-acylphosphatidylethanolamine by members of the phospholipase A/acyltransferase (PLA/AT) family. J. Biol. Chem.

[2] Jin XH, Uyama T, Wang J, Okamoto Y, Tonai T, Ueda N (2009). cDNA cloning and characterization of human and mouse Ca(2+)-independent phosphatidylethanolamine N-acyltransferases. Biochim. Biophys. Acta..

[3] Golczak M, Kiser PD, Sears AE, Lodowski DT, Blaner WS, Palczewski K (2012). Structural basis for the acyltransferase activity of lecithin:retinol acyltransferase-like proteins. J. Biol. Chem.

[4] Anantharaman V, Aravind L (2003). Evolutionary history, structural features and biochemical diversity of the NlpC/P60 superfamily of enzymes. Genome Biol.

[5] Shyu RY, Hsieh YC, Tsai FM, Wu CC, Jiang SY (2008). Cloning and functional characterization of the HRASLS2 gene. Amino acids.

[6] Wei H, Wang L, Ren X, Yu W, Lin J, Jin C (2015). Structural and functional characterization of tumor suppressors TIG3 and H-REV107. FEBS Lett.

[7] Golczak M, Palczewski K (2010). An acyl-covalent enzyme intermediate of lecithin:retinol acyltransferase. J. Biol. Chem.

[8] Shinohara N, Uyama T, Jin XH, Tsuboi K, Tonai T, Houchi H (2011). Enzymological analysis of the tumor suppressor A-C1 reveals a novel group of phospholipid-metabolizing enzymes. J. Lipid Res..

[9] Jin XH, Okamoto Y, Morishita J, Tsuboi K, Tonai T, Ueda N (2007). Discovery and characterization of a Ca2 + −independent phosphatidylethanolamine N-acyltransferase generating the anandamide precursor and its congeners. J. Biol. Chem..

[10] Uyama T, Jin XH, Tsuboi K, Tonai T, Ueda N (2009). Characterization of the human tumor suppressors TIG3 and HRASLS2 as phospholipid-metabolizing enzymes. Biochim. Biophys. Acta..

[11] Hajnal A, Klemenz R, Schafer R (1994). Subtraction cloning of H-rev107, a gene specifically expressed in H-ras resistant fibroblasts. Oncogene.

[12] Uyama T, Ichi I, Kono N, Inoue A, Tsuboi K, Jin XH (2012). Regulation of peroxisomal lipid metabolism by catalytic activity of tumor suppressor H-rev107. J. Biol. Chem.

[13] Jaworski K, Ahmadian M, Duncan RE, Sarkadi-Nagy E, Varady KA, Hellerstein MK (2009). AdPLA ablation increases lipolysis and prevents obesity induced by high-fat feeding or leptin deficiency. Nat. Med..

[14] Duncan RE, Sarkadi-Nagy E, Jaworski K, Ahmadian M, Sul HS (2008). Identification and functional characterization of adipose-specific phospholipase A2 (AdPLA). J. Biol. Chem.

[15] Uyama T, Morishita J, Jin XH, Okamoto Y, Tsuboi K, Ueda N (2009). The tumor suppressor gene H-Rev107 functions as a novel Ca2 + −independent cytosolic phospholipase A1/2 of the thiol hydrolase type. J. Lipid Res..

[16] Pang XY, Cao J, Addington L, Lovell S, Battaile KP, Zhang N (2012). Structure/function relationships of adipose phospholipase A2 containing a cys-his-his catalytic triad. J. Biol. Chem.

[17] Schmid HH, Berdyshev EV (2002). Cannabinoid receptor-inactive N-acylethanolamines and other fatty acid amides: metabolism and function. Prostaglandins Leukot. Essent. Fatty Acids..

[18] Roder K, Latasa MJ, Sul HS (2002). Silencing of the mouse H-rev107 gene encoding a class II tumor suppressor by CpG methylation. J. Biol. Chem.

[19] Roder K, Kim KH, Sul HS (2002). Induction of murine H-rev107 gene expression by growth arrest and histone acetylation: involvement of an Sp1/Sp3-binding GC-box. Biochem. Biophys. Res. Commun..

[20] Roder K, Latasa MJ, Sul HS (2002). Murine H-rev107 gene encoding a class II tumor suppressor: gene organization and identification of an Sp1/Sp3-binding GC-box required for its transcription. B Biochem. Biophys. Res. Commun..

[21] Akiyama H, Hiraki Y, Noda M, Shigeno C, Ito H, Nakamura T (1999). Molecular cloning and biological activity of a novel Ha-Ras suppressor gene predominantly expressed in skeletal muscle, heart, brain, and bone marrow by differential display using clonal mouse EC cells, ATDC5. J. Biol. Chem.

[22] Ito H, Akiyama H, Shigeno C, Nakamura T (2001). Isolation, characterization, and chromosome mapping of a human A-C1 Ha-Ras suppressor gene (HRASLS). Cytogenet. Cell Genet..

[23] Kaneda A, Kaminishi M, Yanagihara K, Sugimura T, Ushijima T (2002). Identification of silencing of nine genes in human gastric cancers. Cancer Res.

[24] Uyama T, Inoue M, Okamoto Y, Shinohara N, Tai T, Tsuboi K (2013). Involvement of phospholipase A/acyltransferase-1 in N-acylphosphatidylethanolamine generation. Biochem. Biophys. Acta..

[25] Nazarenko I, Schafer R, Sers C (2007). Mechanisms of the HRSL3 tumor suppressor function in ovarian carcinoma cells. J. Cell Sci..

[26] Sers C, Emmenegger U, Husmann K, Bucher K, Andres AC, Schafer R (1997). Growth-inhibitory activity and downregulation of the class II tumor-suppressor gene H-rev107 in tumor cell lines and experimental tumors. J. Cell Biol..

[27] Uyama T, Kawai K, Kono N, Watanabe M, Tsuboi K, Inoue T (2015). Interaction of Phospholipase A/Acyltransferase-3 with Pex19p: A POSSIBLE INVOLVEMENT IN THE DOWN-REGULATION OF PEROXISOMES. J Biol. Chem.

[28] Yanatatsaneejit P, Chalermchai T, Kerekhanjanarong V, Shotelersuk K, Supiyaphun P, Mutirangura A (2008). Promoter hypermethylation of CCNA1, RARRES1, and HRASLS3 in nasopharyngeal carcinoma. Oral oncol.

[29] Nazarenko I, Kristiansen G, Fonfara S, Guenther R, Gieseler C, Kemmner W (2006). H-REV107-1 stimulates growth in non-small cell lung carcinomas via the activation of mitogenic signaling. Am. J. Pathol..

[30] Shyu RY, Wu CC, Wang CH, Tsai TC, Wang LK, Chen ML (2013). H-rev107 regulates prostaglandin D2 synthase-mediated suppression of cellular invasion in testicular cancer cells. J. Biomed. Sci..

[31] Wang CH, Shyu RY, Wu CC, Tsai TC, Wang LK, Chen ML (2014). Phospholipase A/Acyltransferase enzyme activity of H-rev107 inhibits the H-RAS signaling pathway. J. Biomed. Sci..

[32] Xiong S, Tu H, Kollareddy M, Pant V, Li Q, Zhang Y (2014). Pla2g16 phospholipase mediates gain-of-function activities of mutant p53. Proc. Natl. Acad. Sci. U. S. A..

[33] Ren X, Lin J, Jin C, Xia B (2010). Solution structure of the N-terminal catalytic domain of human H-REV107--a novel circular permutated NlpC/P60 domain. FEBS Lett.

[34] Narumiya S, Sugimoto Y, Ushikubi F (1999). Prostanoid receptors: structures, properties, and functions. Physiol. Rev..

[35] DiSepio D, Ghosn C, Eckert RL, Deucher A, Robinson N, Duvic M (1998). Identification and characterization of a retinoid-induced class II tumor suppressor/growth regulatory gene. Proc. Natl. Acad. Sci. U. S. A..

[36] Huang SL, Shyu RY, Yeh MY, Jiang SY (2000). Cloning and characterization of a novel retinoid-inducible gene 1(RIG1) deriving from human gastric cancer cells. Mol. Cell. Endocrinol..

[37] Casanova B, de la Fuente MT, Garcia-Gila M, Sanz L, Silva A, Garcia-Marco JA (2001). The class II tumor-suppressor gene RARRES3 is expressed in B cell lymphocytic leukemias and down-regulated with disease progression. Leukemia.

[38] Hsu TH, Chu CC, Jiang SY, Hung MW, Ni WC, Lin HE (2012). Expression of the class II tumor suppressor gene RIG1 is directly regulated by p53 tumor suppressor in cancer cell lines. FEBS Lett.

[39] Tsai FM, Shyu RY, Jiang SY (2007). RIG1 suppresses Ras activation and induces cellular apoptosis at the Golgi apparatus. Cellular signalling.

[40] Huang SL, Shyu RY, Yeh MY, Jiang SY (2002). The retinoid-inducible gene I: effect on apoptosis and mitogen-activated kinase signal pathways. Anticancer Res.

[41] Morales M, Arenas EJ, Urosevic J, Guiu M, Fernandez E, Planet E (2014). RARRES3 suppresses breast cancer lung metastasis by regulating adhesion and differentiation. EMBO Mol. Med..

[42] Wu CC, Shyu RY, Wang CH, Tsai TC, Wang LK, Chen ML (2012). Involvement of the prostaglandin D2 signal pathway in retinoid-inducible gene 1 (RIG1)-mediated suppression of cell invasion in testis cancer cells. Biochim. Biophys. Acta..

[43] Scharadin TM, Eckert RL (2014). TIG3: an important regulator of keratinocyte proliferation and survival. J. Invest. Dermatol.

[44] Jans R, Sturniolo MT, Eckert RL (2008). Localization of the TIG3 transglutaminase interaction domain and demonstration that the amino-terminal region is required for TIG3 function as a keratinocyte differentiation regulator. J. Invest. Dermatol.

[45] Sturniolo MT, Dashti SR, Deucher A, Rorke EA, Broome AM, Chandraratna RA (2003). A novel tumor suppressor protein promotes keratinocyte terminal differentiation via activation of type I transglutaminase. J. Biol. Chem.

[46] Sturniolo MT, Chandraratna RA, Eckert RL (2005). A novel transglutaminase activator forms a complex with type 1 transglutaminase. Oncogene.

[47] Duvic M, Helekar B, Schulz C, Cho M, DiSepio D, Hager C (2000). Expression of a retinoid-inducible tumor suppressor, Tazarotene-inducible gene-3, is decreased in psoriasis and skin cancer. Clin. Cancer Res..

[48] Scharadin TM, Adhikary G, Shaw K, Grun DJ, Xu W, Eckert RL (2014). Pericentrosomal localization of the TIG3 tumor suppressor requires an N-terminal hydrophilic region motif. J. Invest. Dermatol.

[49] Yamano Y, Asano A, Ohyama K, Ohta M, Nishio R, Morishima I (2008). Expression of the Ha-ras suppressor family member 5 gene in the maturing rat testis. Biosci. Biotechnol. Biochem..

[50] Vaccari CM, Romanini MV, Musante I, Tassano E, Gimelli S, Divizia MT (2014). De novo deletion of chromosome 11q12.3 in monozygotic twins affected by Poland Syndrome. BMC Med. Genet..

[51] Larkin MA, Blackshields G, Brown NP, Chenna R, McGettigan PA, McWilliam H (2007). ClustalW and ClustalX version 2. Bioinformatics.

